# Accuracy of ICD Influenza Discharge Diagnosis Codes in Hospitalized Adults From the Valencia Region, Spain, in the Pre‐COVID‐19 Period 2012/2013 to 2017/2018

**DOI:** 10.1111/irv.70069

**Published:** 2025-02-05

**Authors:** Ainara Mira‐Iglesias, Mónica López‐Lacort, Hélène Bricout, Matthew Loiacono, Mario Carballido‐Fernández, Joan Mollar‐Maseres, Miguel Tortajada‐Girbés, Germán Schwarz‐Chávarri, F. Xavier López‐Labrador, Joan Puig‐Barberà, Javier Díez‐Domingo, Alejandro Orrico‐Sánchez

**Affiliations:** ^1^ Área de Investigación en Vacunas Fundación Para el Fomento de la Investigación Sanitaria y Biomédica de la Comunitat Valenciana (FISABIO‐Public Health) Valencia Spain; ^2^ CIBER de Epidemiología y Salud Pública Instituto de Salud Carlos III Madrid Spain; ^3^ Sanofi Lyon France; ^4^ Sanofi Swiftwater Pennsylvania USA; ^5^ Hospital General Universitario de Castellón Castellón de la Plana Spain; ^6^ Universidad CEU Cardenal Herrera Castellón de la Plana Spain; ^7^ Hospital Universitario y Politécnico La Fe Valencia Spain; ^8^ Hospital Universitario Doctor Peset Valencia Spain; ^9^ Hospital General Universitario de Alicante Alicante Spain; ^10^ Laboratorio de Virología, Área de Genómica y Salud Fundación Para el Fomento de la Investigación Sanitaria y Biomédica de la Comunitat Valenciana (FISABIO‐Public Health) Valencia Spain; ^11^ Universidad Católica de Valencia San Vicente Mártir Valencia Spain

**Keywords:** active surveillance, diagnosis codes, discharge diagnoses, hospitalizations, ICD codes, influenza, influenza‐like illness, laboratory‐confirmed influenza

## Abstract

**Background:**

International Classification of Diseases (ICD) codes obtained from real‐world data can be used to identify influenza cases for epidemiological research but, without validation, may introduce biases. The objective of this study was to validate ICD influenza discharge diagnoses using real‐time reverse transcription‐polymerase chain reaction (RT‐PCR) laboratory‐confirmed influenza (LCI) results.

**Methods:**

The study was conducted during six influenza seasons (2012/2013–2017/2018) in the Valencia Hospital Surveillance Network for the Study of Influenza (VAHNSI). Patients aged 18+ years were identified via active‐surveillance and had to meet an influenza‐like illness (ILI) case definition to be included. All patients were tested for influenza by real‐time RT‐PCR. Main and secondary influenza discharge diagnosis codes were extracted from hospital discharge letters. Positive predictive values (PPVs) and the complementary of the sensitivities (1‐Sensitivity) of ICD codes with corresponding 95% credible intervals (CrIs) were estimated via binomial Bayesian regression models.

**Results:**

A total of 13,545 patients were included, with 2257 (17%) positive for influenza. Of 2257 LCI cases, 1385 (61%) were not ICD‐coded as influenza. Overall, 74.73% (95% CrI: 63.24–84.44) of LCI were not‐ICD coded as influenza (1‐Sensitivity) after adjustment. Sensitivity improved across seasons and with increasing age. Average PPV was 74.02% (95% CrI: 68.58–79.17), ranging from 43.71% to 81.57% between seasons.

**Conclusion:**

Using only main and secondary discharge diagnosis codes for influenza detection markedly underestimates the full burden of influenza in hospitalized patients. Future studies, including post‐COVID context, using prospective surveillance for ILI are required to assess the validity of hospital discharge data as a tool for determining influenza‐related burden of disease.

## 1 Background

Influenza represents a significant public health burden in high income countries. Annually, it accounts for 3 to 5 million severe cases, with 290,000 to 650,000 deaths worldwide [1, 2]. Precise estimates of the impact of influenza on hospital systems are essential at both regional and national levels to optimize resource allocation and assess the cost‐effectiveness of targeted interventions, such as vaccination strategies. Significant resources have been used to carry out studies to estimate the burden of influenza and its prevention [3, 4, 5, 6].

International Classification of Diseases (ICD) codes obtained from the clinical practice routine (real world data, RWD) could be used as a tool to identify influenza cases for epidemiological research. However, with influenza and other respiratory infections potentially producing similar clinical manifestations, it is challenging to ascertain an influenza‐specific diagnosis in the absence of laboratory confirmation testing [7]. Although reverse transcription‐polymerase chain reaction (RT‐PCR) remains the gold standard for influenza diagnosis, other tests such as immunochromatography or immunofluorescence with lower sensitivity have been commonly used in clinical practice [8, 9]. Moreover, several aspects such as the seasonal nature of the influenza and the clinical judgement of doctors and clinical coders could further interfere with the sensitivity and predictive values of ICD codes [10].

Therefore, using ICD codes, without having validated their accuracy, may introduce biases in influenza case ascertainment. For this reason, the objective of this study was to validate ICD influenza discharge diagnoses using gold standard RT‐PCR laboratory‐confirmed influenza (LCI) results obtained from an independent active‐surveillance hospital network for the study of influenza and other respiratory viral infections.

## 2 Methods

### 2.1 Study Design

This is a retrospective analysis of prospectively collected data during six pre‐COVID‐19 pandemic influenza seasons, from 2012/2013 to 2017/2018, within the framework of the Valencia Hospital Surveillance Network for the Study of Influenza (VAHNSI) [11, 12, 13]. Depending on the season, 4 to 10 of the following hospitals from the Valencia Region participated in the prospective active influenza surveillance and data collection (Table S1). Collectively, these hospitals provided healthcare to 21% (4 hospitals) to 46% (10 hospitals) of the inhabitants of the Valencia Region. The study was conducted annually from November to March/April.

**FIGURE 1:**
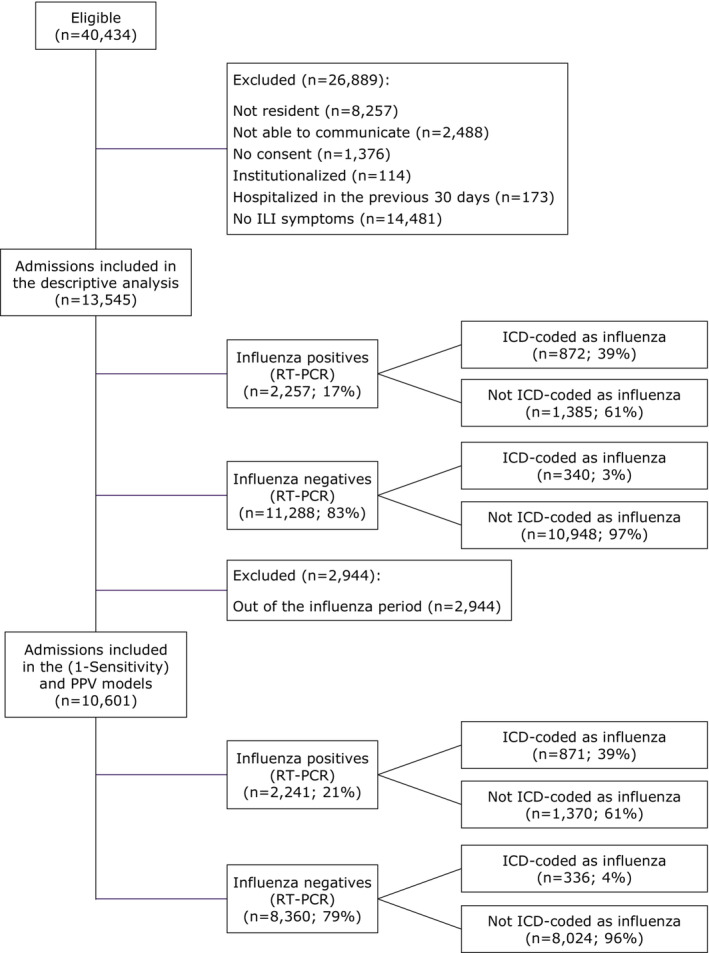
Participant disposition flow diagram. ICD, International Classification of Diseases; ILI, influenza‐like illness; PPV, positive predictive value; RT‐PCR, real‐time reverse transcription‐polymerase chain reaction.

The surveillance methodology has been described previously [11, 12, 13]. Full‐time dedicated nurses screened consecutive hospitalized patients admitted from the Emergency Department with a diagnosis possibly related to influenza. Patients were included in the study if they were resident in the catchment area of one of the participating hospitals, noninstitutionalized, not discharged from a previous admission in the last 30 days and provided written‐informed consent. Patients had to meet the European Centre for Disease Prevention and Control (ECDC) influenza‐like illness (ILI) case definition [14], defined as the presence of at least one systemic symptom (fever or feverishness, malaise, myalgia or headache) and at least one respiratory symptom (shortness of breath, sore throat or cough). Onset of symptoms was required in the 7 days prior to admission and patients had to be hospitalized between 8 and 48 h before their inclusion in the study to ensure that patients spent at least one night in the hospital and to avoid the inclusion of potential nosocomial infections. The current analysis was restricted to patients 18 years old or over (Figure 1).

### 2.2 RT‐PCR LCI (Gold Standard) and ICD Influenza Discharge Diagnosis Codes

All included patients were tested for influenza by real‐time RT‐PCR taking our own nasopharyngeal sample collected and analysed in the same laboratory following the same procedures, and those testing positive for influenza were considered as LCI cases. Clinicians in charge of the patients and study field investigators were unaware of the RT‐PCR result, as this was only used for research purposes. Main and secondary discharge diagnoses were directly extracted from the discharge letters codified by hospital clinicians. When more than one secondary diagnosis at discharge was codified, the most closely related to influenza or its complications was selected. We considered that a patient was coded for influenza if his/her main or secondary discharge diagnoses were 487–488 (ICD9) or J09‐J11 (ICD10). ICD9 codification was used until 2016. Afterwards, the use of ICD10 codification was established.

### 2.3 Laboratory Procedures

Two different swabs, nasopharyngeal and pharyngeal (FLOQSwabs, Copan, Italy), were obtained within the first 8–48 h of admission from all patients fulfilling the inclusion criteria. Both swabs were combined in one tube of viral transport media (Copan, Italy) and frozen until shipped refrigerated to a centralized Virology laboratory at FISABIO‐Public Health. One third of the viral transport media volume was extracted for total nucleic acids using an automated silica‐based method (Nuclisens Easy‐Mag, BioMérieux, Lyon, France) and tested for influenza viruses by multiplex real‐time RT‐PCR, following WHO protocols [15] with the qScript XLT One‐Step RT‐qPCR ToughMix (Quanta BioSciences, MD, USA) in a Lightcycler 480II apparatus (Roche Diagnostics, Spain). First, a real‐time RT‐PCR assay was performed to detect and differentiate influenza A and B viruses using different primers and probes for the matrix protein [16]. Thereafter, two different real‐time RT‐PCR typing assays were performed to determine the viral subtype/lineage of influenza A or B viruses on influenza‐positive samples [17, 18].

### 2.4 Statistical Analysis

The distribution of LCI cases and their corresponding main and secondary discharge diagnoses ICD‐coded (differentiating between circulatory and respiratory system diseases, some acute cardiovascular events and pneumonia; Table S2 for more detail) were summarized overall, by age and season using frequencies and proportions.

Considering PCR results as gold standard, we estimated the probability of not being coded as influenza when the PCR result was positive (1‐Sensitivity) using a binomial Bayesian regression model including the season as a covariate and hospital as a random effect (to account the variability among hospitals). All patients with LCI identified in our surveillance system were considered. The outcome variable in this case was the ICD code (0 = *not coded as influenza* and 1 = *coded as influenza*). Overall, 1‐Sensitivity was estimated including the intercept and a random effect for the interaction term between hospital and season.

The influenza ICD codes' positive predictive value (PPV) was defined as the probability of being a LCI if the ICD code referred to influenza. All patients identified in our surveillance system with an ICD code for influenza in the main or secondary discharge diagnoses were considered to estimate the PPV values. We estimated PPV by season as a covariate, hospital as a random effect and the influenza PCR result (0 = *negative for influenza* and 1 = *positive for influenza*) as response variable. Overall PPV was estimated throughout a binomial Bayesian regression including the intercept and a random effect for the interaction term between hospital and season.

We estimated both 1‐Sensitivity and the PPV and their 95% credibility intervals (CrI), overall and by age group (18–49, 50–59, 60–74 and ≥ 75 years). For both models, patients admitted out of the influenza season were excluded (Figure 1).

## 3 Results

### 3.1 Study Population

We included in the study and tested a total of 13,545 patients 18 years old or over. Of all patients, 1380 (10.19%) were 18–49 years old, 2084 (15.39%) were 50–64, 2803 (20.69%) were 65–74 and 7278 (53.73%) were 75 years old or over. A total of 2257 (16.66%) samples tested positive for influenza (Table S3). Similar proportions of LCI occurred in the different age groups, being higher in those 65–74 (Table S4). A description of the influenza strains circulation by season and age is also provided in Tables S3 and S4.

### 3.2 LCI Cases According to Main and Secondary Discharge Diagnoses (ICD Code)

Of the total 2257 LCI cases observed in the surveillance system, 1385 (61.4%) were not ICD‐coded as influenza. Out of those not ICD‐coded as influenza cases, 59.6% were discharged with either primary or secondary ICD‐coded diagnoses related to respiratory system diseases, and 11.1% were related to circulatory system diseases. Subjects without an ICD‐coded influenza diagnosis had a higher percentage of pneumonia cases compared to those with ICD diagnoses, especially in the oldest age group (5.6% vs. 21.3%). The same pattern was observed in subjects aged 50–64, 65–74 and 75 years or older with circulatory system‐related diseases (Table 1).

**TABLE 1:** Laboratory‐confirmed influenza (LCI) cases by main and secondary ICD discharge diagnoses code.

	Overall (*N* = 2257)		18–49 year (*N* = 226)		50–64 year (*N* = 341)		65–74 year (*N* = 511)		≥75 year (*N* = 1179)	
	*N* (%)		*N* (%)		*N* (%)		*N* (%)		*N* (%)	
	ICD‐I	No ICD‐I	ICD‐I	No ICD‐I	ICD‐I	No ICD‐I	ICD‐I	No ICD‐I	ICD‐I	No ICD‐I
LCI	872 (38.6%)	1385 (61.4%)	112 (49.6%)	114 (50.4%)	148 (43.4%)	193 (56.6%)	216 (42.3%)	295 (57.7%)	396 (33.6%)	783 (66.4%)
Circulatory system‐related disease	24 (2.8%)	154 (11.1%)	0 (0%)	1 (0.9%)	5 (3.4%)	14 (7.3%)	8 (3.7%)	38 (12.9%)	11 (2.8%)	101 (12.9%)
Hypertension	0 (0%)	3 (0.2%)	0 (0%)	0 (0%)	0 (0%)	1 (0.5%)	0 (0%)	0 (0%)	0 (0%)	2 (0.3%)
Ischemic heart disease	2 (0.2%)	29 (2.1%)	0 (0%)	0 (0%)	0 (0%)	6 (3.1%)	2 (0.9%)	10 (3.4%)	0 (0%)	13 (1.7%)
Pulmonary heart disease	1 (0.1%)	6 (0.4%)	0 (0%)	0 (0%)	0 (0%)	1 (0.5%)	1 (0.5%)	2 (0.7%)	0 (0%)	3 (0.4%)
Other forms of heart disease	21 (2.4%)	114 (8.2%)	0 (0%)	1 (0.9%)	5 (3.4%)	6 (3.1%)	5 (2.3%)	26 (8.8%)	11 (2.8%)	81 (10.3%)
Cerebrovascular disease	0 (0%)	5 (0.4%)	0 (0%)	1 (0.9%)	0 (0%)	0 (0%)	0 (0%)	0 (0%)	0 (0%)	4 (0.5%)
Diseases of arteries	0 (0%)	4 (0.3%)	0 (0%)	0 (%)	0 (0%)	1 (0.5%)	0 (0%)	0 (0%)	0 (0%)	3 (0.4%)
Respiratory system‐related disease	872 (100%)	826 (59.6%)	112 (100%)	75 (65.8%)	148 (100%)	132 (68.4%)	216 (100%)	187 (63.4%)	396 (100%)	432 (55.2%)
Acute upper respiratory infections	1 (0.1%)	58 (4.2%)	0 (0%)	8 (7%)	0 (0%)	7 (3.6%)	0 (0%)	19 (6.4%)	1 (0.3%)	24 (3.1%)
Influenza/pneumonia	872 (100%)	298 (21.5%)	112 (100%)	35 (30.7%)	148 (100%)	39 (20.2%)	216 (100%)	57 (19.3%)	396 (100%)	167 (21.3%)
Other acute lower respiratory infections	7 (0.8%)	143 (10.3%)	1 (0.9%)	5 (4.4%)	0 (0%)	16 (8.3%)	2 (0.9%)	26 (8.8%)	4 (1%)	96 (12.3%)
Chronic lower respiratory disease	83 (9.5%)	346 (25%)	16 (14.3%)	30 (26.3%)	17 (11.5%)	73 (37.8%)	29 (13.4%)	95 (32.2%)	21 (5.3%)	148 (18.9%)
Other respiratory diseases (interstitium)	18 (2.1%)	46 (3.3%)	2 (1.8%)	3 (2.6%)	6 (4.1%)	8 (4.1%)	3 (1.4%)	9 (311%)	7 (1.8%)	26 (3.3%)
Suppurative and necrotic conditions of the lower respiratory tract	0 (0%)	2 (0.1%)	0 (0%)	1 (0.9%)	0 (0%)	1 (0.5%)	0 (0%)	0 (0%)	0 (0%)	0 (0%)
Selected potential influenza‐related complications										
Pneumonia	80 (9.2%)	298 (21.5%)	17 (15.2%)	35 (30.7%)	16 (10.8%)	39 (20.2%)	25 (11.6%)	57 (19.3%)	22 (5.6%)	167 (21.3%)
Heart failure	14 (1.6%)	90 (6.5%)	0 (0%)	1 (0.9%)	2 (1.4%)	5 (2.6%)	2 (0.9%)	20 (6.8%)	10 (2.5%)	64 (8.2%)
Acute myocardial infarction	1 (0.1%)	7 (0.5%)	0 (0%)	0 (0%)	0 (0%)	1 (0.5%)	1 (0.5%)	2 (0.7%)	0 (0%)	4 (0.5%)
Atrial fibrillation	4 (0.5%)	21 (1.5%)	0 (0%)	0 (0%)	2 (1.4%)	1 (0.5%)	2 (0.9%)	6 (2.0%)	0 (0%)	14 (1.8%)
Stroke	0 (0%)	2 (0.1%)	0 (0%)	0 (0%)	0 (0%)	0 (0%)	0 (0%)	0 (0%)	0 (0%)	2 (0.3%)

*Note:* Data stratified by presence or absence of influenza (ICD‐I) codes and age. Hospitalized adults (≥ 18 years old) from the Valencia Hospital Surveillance Network (VAHNSI), Valencia Region, Spain (2012/2013 to 2017/2018). The columns do not add up the totals since the diagnoses are not exclusive (we are considering the primary and secondary).

Abbreviations: ICD‐I, International Classification of Diseases‐Influenza; LCI, laboratory‐confirmed influenza.

### 3.3 LCI Cases Not ICD‐Coded as Influenza at Discharge

The percentages of LCI not ICD‐coded as influenza (1‐Sensitivity) by season and age are shown in Table 2. Overall, 74.73% (95% CrI: 63.24–84.44) of LCI were not ICD‐coded as influenza after adjustment. The sensitivity of the ICD‐codes improved throughout the seasons overall and by age group, reducing the underestimation of influenza from 96.68% to 41.91%. The sensitivity of influenza‐related ICD code was similar for patients between 18 and 74 years old. The highest percentage of influenza diagnosis underestimation was found in subjects over 74 years.

**TABLE 2:** Percentage of LCI not ICD‐coded as influenza^a^ by season, age and overall.^b^

1‐Sensitivity (95% CrI)					
Season	Overall	18–49 years	50–64 years	65–74 years	≥ 75 years
12/13	96.7	88.2	91.6	93.6	99.4
	(92.0–99.1)	(71.4–98.0)	(75.1–99.0)	(82.8–98.9)	(97.4–100.0)
13/14	68.8	42.6	52.2	48.4	88.7
	(47.9–85.7)	(25.9–59.5)	(31.7–72.3)	(31.1–69.1)	(74.2–97.8)
14/15	82.6	62.1	79.2	74.7	87.2
	(65.8–92.5)	(43.0–79.6)	(62.8–91.2)	(60.3–86.4)	(73.6–97.0)
15/16	79.0	62.9	58.0	75.9	87.0
	(58.6–91.5)	(43.6–81.9)	(35.0–79.4)	(58.6–90.1)	(71.2–97.3)
16/17	58.0	33.3	51.1	45.8	64.0
	(35.1–78.1)	(11.7–62.1)	(25.0–79.8)	(25.9–68.5)	(38.1–89.1)
17/18	41.9	17.2	26.3	30.2	50.9
	(21.3–63.5)	(6.4–32.0)	(12.8–46.1)	(17.4–48.7)	(26.2–82.4)

Abbreviations: CrI, credibility interval; ICD, International Classification of Diseases; LCI, laboratory‐confirmed influenza.

^a^ Main and secondary diagnoses (i.e., 1‐Sensitivity).

^b^ Hospitalized adults (≥ 18 years old) from the Valencia Hospital Surveillance Network (VAHNSI), Valencia Region, Spain (2012/2013 to 2017/2018).

### 3.4 Positive Predictive Values (PPV) of ICD Codes Related to Influenza

The estimated PPVs of influenza‐related ICD codes, either main or secondary discharge diagnoses, by season and age are shown in Table 3. The average PPV was 74.02% (95% CrI: 68.58–79.17), ranging from 43.71% to 81.57% between seasons. Overall, PPV estimates were consistent across the age groups within each season.

**TABLE 3:** Positive predictive value and 95% credibility intervals of influenza ICD codification by season, age and overall.^a^

PPV (95% CrI)					
Season	Overall	18–49 years	50–64 years	65–74 years	≥ 75 years
12/13	43.7	43.8	35.9	72.4	32.9
	(21.7–67.8)	(9.8–82.7)	(4.8–79.6)	(18.8–99.4)	(1.0–82.1)
13/14	73.4	70.4	69.4	85.9	79.8
	(62.2–82.9)	(51.4–88.7)	(46.5–87.2)	(66.5–96.9)	(62.2–93.5)
14/15	81.6	76.2	76.5	86.7	82.3
	(72.9–88.5)	(49.5–95.0)	(51.8–93.4)	(69.9–96.9)	(71.8–90.0)
15/16	74.7	67.4	80.9	82.9	72.1
	(61.0–84.8)	(35.2–88.4)	(56.1–94.7)	(56.6–96.8)	(53.7–86.8)
16/17	57.4	61.0	27.4	71.5	66.0
	(44.2–68.9)	(30.9–84.6)	(7.9–53.1)	(41.8–91.2)	(51.9–77.9)
17/18	71.2	70.3	63.1	76.2	76.6
	(59.3–80.2)	(47.2–87.1)	(38.8–81.0)	(54.4–92.9)	(68.4–84.4)

Abbreviations: CrI, credibility interval; ICD, international Classification of Diseases; PPV, Positive Predictive Value.

^a^ Hospitalized adults (≥ 18 years old) from the Valencia Hospital Surveillance Network (VAHNSI), Valencia Region, Spain (2012/2013 to 2017/2018).

## 4 Discussion

In our study, we estimated that before the COVID‐19 pandemic, there was a high percentage of LCI not ICD‐coded as influenza, though improved over time. A moderate PPV of ICD influenza discharge diagnosis codes was found among patients 18 years and over. The improvement in the influenza codification over time was possibly due to the increase usage of diagnostic tests and wider use of the PCR in clinical practice. This increasing integration of multiplex RT‐PCR tests in routine allows to detect influenza and differentiate it from other pathogens, reducing diagnostic misclassification due to its improved specificity and sensitivity compared to rapid antigen detection tests [19, 20, 21]. Also, there might be a refinement in the clinical codification of the cases as protocols and training for ICD coding have improved over time [21]. The PPV showed a notable decline in the 2016/2017 season, possibly due to the change from ICD‐9‐CM to ICD‐10‐CM coding in January of 2016. No relevant differences were observed with age groups but among seasons.

Our findings were comparable to those previously published. In the United States (US), Elkin et al. [9] reported a PPV of 66% and a sensitivity of 46%. However, the study was limited to data from a single large academic medical institution, which may limit its generalizability. Similarly, a PPV of 60% and a higher sensitivity of 70% were described for influenza‐specific ICD codes in a study by Feemster et al. [22] in the US. These estimations were obtained only for paediatric population, and similar to Elkin et al. [9], no adjustment for confounding variables was performed [22]. In Australia, Moore et al. [23] found higher values for both PPV and sensitivity, 84% and 86%, respectively in children ≤ 9 years old. Unlike our study, they included blood and pleural fluid samples as well. Additionally, their gold standard for diagnosis involved not only PCR but also immunofluorescence and culture methods. Keren et al. [8] conducted a study in a single hospital involving subjects under 21 years old. They reported a sensitivity of 65% (95% CI: 61–68%) and a PPV of 88% (95% CI: 84–90%). The diagnostic methods included rapid immunoassay, direct fluorescent antibody test and viral culture. Rapid influenza tests with imperfect specificity may result in a small but significant number of false‐positive results [8]. Unlike the other studies, we included all codes related to influenza (487.XX‐488.XX and J09.XX‐J11.XX). Moore et al. [23] did not include the ICD‐10 J09.XX code, whereas Elkin et al. [9], Feemster et al. [22] and Keren et al. [8] only evaluated the ICD‐9487.XX code. Other authors have also found strong correlations between particular ICD‐10 codes (J11, J06, J22, B34 and J18) and influenza diagnosis in small Australian samples and during peaks of epidemic activity, such as that which occurred in 2009 [10].

Several factors may influence influenza diagnosis coding practices. The different hospitals' policies (among them and over time) and the clinicians' perception of influenza circulation, as well as the cocirculation of other respiratory viruses, may influence their likelihood of diagnosing an influenza infection [8, 10]. Differential utilization of rapid tests and their varying specificity and sensitivity may also lead to underestimation of the influenza hospitalizations [8].

The results of our work were generated in the pre‐COVID‐19 pandemic period, when swabbing and testing practices have been widely used [24]. It is expected that these practices remain in hospitals and the codification of influenza improve. Future research in the post‐COVID‐19 era is needed to assess if and how such practices have changed.

The underestimation of influenza burden may lead to undervaluing the risk of developing the disease, which is particularly concerning for more vulnerable populations. Therefore, it may cast doubt on the importance of influenza vaccination or its cost‐effectiveness. Conversely, obtaining more accurate estimates can highlight the necessity and impact of vaccination, especially in reducing severe cases of infection in at‐risk groups. Moreover, this would allow public health economic resources to be allocated more efficiently and contribute to the improvement of vaccine recommendations.

The main strength of this study was the concomitance of prospective active influenza surveillance in a sample of hospitals from the Valencia Region of Spain and the availability and accessibility to hospital ICD discharge diagnoses registered in the Valencia Health System Integrated Database [25]. Furthermore, it is essential to highlight that our ICD‐codes remained uninfluenced by the gold standard, as the clinicians conducting the study were unaware of the PCR results, ensuring no contamination or bias in the study. Consequently, we were able to work on a large sample size as well as on a range of seasons. That fact allowed us to adjust our estimates by hospital and season. Moreover, across the six analysed seasons, all samples followed the same process and were tested by real‐time RT‐PCR in a centralized laboratory in FISABIO.

Some studies on this topic have been based in individual seasons or used aggregated data over time, rendering it challenging to disentangle changes in reporting, medical practices or hospital policies that may bias PPV and sensitivity estimates [22]. Therefore, caution should be taken when interpreting these former analyses, as their results may be either overestimated or underestimated. By contrast, we estimated the PPV and the proportion of LCI not ICD‐coded while adjusting for season and accounting for variability among hospitals, thereby improving the robustness of our findings.

Our analysis of the concordance between laboratory tests and ICD codes helps to address an important evidence gap, as it allows us to better understand the degree to which the diagnosis and burden of influenza may be underestimated.

Frequently, the aetiology confirmation of different viral infections does not affect the treatment or clinical practice. Therefore, more unspecific codes can be reported, leading to underestimating the full ICD‐coded influenza burden of disease. Although it is not a limitation itself, we studied the PPV and the percentage of LCI not ICD‐coded as influenza considering overall influenza. That choice was justified given our objectives, but one could consider further exploring the possibility of obtaining those estimations by strain/lineage‐specific influenza‐related ICD codes. Lastly, the frequency of ICD‐coded influenza‐related complications we observed, such as those related to the circulatory system or pneumonia, likely underestimate of the full burden of influenza [26, 27]. Influenza could be diagnosed in diagnostic positions other than the primary and secondary positions, due to the presence of these more severe diagnoses. These complications may occur later after the initial infection, when influenza‐related symptoms are less prominent, and thus, these patients would not have been captured by the surveillance system and included in our study.

## 5 Conclusion

Influenza‐related ICD‐codes are insufficient for influenza surveillance in hospitalized patients. Our results demonstrated that using clinical practice routine data to detect LCI cases has low sensitivity but moderate PPV. Relying on the presence of an influenza‐specific ICD code may lead to underestimating the true percentage of LCI cases among hospitalized patients. By contrast, the lack of a discharge code for influenza cannot reliably indicate the absence of a laboratory confirmed influenza infection.

In conclusion, using only main and secondary discharge diagnosis codes for influenza detection markedly underestimates the full burden of influenza in hospitalized patients. Future studies, including post‐COVID context, using prospective surveillance for ILI are required to assess the validity of hospital discharge data as a tool for determining influenza‐related burden of disease.

## Author Contributions

**Ainara Mira‐Iglesias:** conceptualization, data curation, validation, formal analysis, writing – original draft. **Mónica López‐Lacort:** conceptualization, formal analysis, validation, writing – original draft. **Hélène Bricout:** writing – review and editing. **Matthew Loiacono:** writing – review and editing. **Mario Carballido‐Fernández:** investigation, writing – review and editing. **Joan Mollar‐Maseres:** investigation, writing – review and editing. **Miguel Tortajada‐Girbés:** investigation, writing – review and editing. **Germán Schwarz‐Chávarri:** investigation, writing – review and editing. **F. Xavier López‐Labrador:** data curation, validation, writing – review and editing. **Joan Puig‐Barberà:** writing – review and editing. **Javier Díez‐Domingo:** conceptualization, validation, supervision, writing – original draft. **Alejandro Orrico‐Sánchez:** writing – review and editing.

## Ethics Statement

The Ethics Research Committee of the Dirección General de Salud Pública‐Centro Superior de Investigación en Salud Pública (DGSP‐CSISP) approved the VAHNSI protocol. All patients signed the written‐informed consent prior to their inclusion in the study, which was conducted in accordance with the principles of the Declaration of Helsinki.

## Conflicts of Interest

AMI has received fees for conferences/experts' meetings from Sanofi and for educational events from MSD. MLL has received fees for conferences meetings from Sanofi and for educational events from MSD. MLL has attended to several congresses whose registration, travel and accommodation costs have been covered by MSD, AZ and Sanofi. HB and ML are employees of Sanofi and may hold shares and/or stock options in the company. JDD has attended to several congresses whose registration, travel and accommodation costs have been covered by MSD, GSK and Sanofi. JDD and his institution received research grants from Sanofi and GSK related to RSV preventive strategies. JDD acted as advisor for these immunization strategies to Sanofi. AOS has attended to several congresses whose registration, travel and accommodation costs have been covered by MSD, GSK, Novavax and Sanofi. AOS and his institution received research grants from Sanofi and MSD related to RSV preventive strategies. AOS acted as advisor for these immunization strategies to Sanofi and Moderna. FXLL, MCF, MTG, JMM, GSC and JPB declare no conflicts of interest.

### Peer Review

The peer review history for this article is available at https://www.webofscience.com/api/gateway/wos/peer‐review/10.1111/irv.70069.

## Supporting information


**Table S1.** Participating hospitals in the Valencia Hospital Surveillance Network (VAHNSI), Valencia Region, Spain (2012/2013 to 2017/2018).
**Table S2.** ICD9 and ICD10 codes according to the International Statistical Classification of Diseases and Related Health Problems 10^th^ Revision of the World Health Organization considered for reporting main and secondary discharge diagnoses.
**Table S3.** Included subjects and laboratory‐confirmed influenza (LCI) cases by season and overall. Hospitalized adults (≥18 years old) from the Valencia Hospital Surveillance Network (VAHNSI), Valencia Region, Spain (2012/2013 to 2017/2018).
**Table S4.** Included subjects (18+) and laboratory‐confirmed influenza (LCI) cases by age and overall. Hospitalized adults (≥18 years old) from the Valencia Hospital Surveillance Network (VAHNSI), Valencia Region, Spain (2012/2013 to 2017/2018).

## Data Availability

Data are available upon reasonable request to the corresponding author.
