# Supplementary material for: Accuracy of ICD Influenza Discharge Diagnosis Codes in Hospitalized Adults From the Valencia Region, Spain, in the Pre‐COVID‐19 Period 2012/2013 to 2017/2018
Source: Influenza Other Respir Viruses. 2025 Feb 5;19(2):e70069. doi: 10.1111/irv.70069 (PMC11798732; doi:10.1111/irv.70069)
Supplement: Supplementary file 1 — Table S1. Participating hospitals in the Valencia Hospital Surveillance Network (VAHNSI), Valencia Region, Spain (2012/2013 to 2017/2018). Table S2. ICD9 and ICD10 codes according to the International Statistical Classification of Diseases and Related Health Problems 10th Revision of the World Health Organization considered for reporting main and secondary discharge diagnoses. Table S3. Included subjects and laboratory‐confirmed influenza (LCI) cases by season and overall. Hospitalized adults (≥18 years old) from the Valencia Hospital Surveillance Network (VAHNSI), Valencia Region, Spain (2012/2013 to 2017/2018). Table S4. Included subjects (18+) and laboratory‐confirmed influenza (LCI) cases by age and overall. Hospitalized adults (≥18 years old) from the Valencia Hospital Surveillance Network (VAHNSI), Valencia Region, Spain (2012/2013 to 2017/2018). [file IRV-19-e70069-s001.docx]

**Supplementary Table S1.** Participating hospitals in the Valencia Hospital Surveillance Network (VAHNSI), Valencia Region, Spain (2012/2013 to 2017/2018).

| **Hospitals** | **2012/2013** | **2013/2014** | **2014/2015** | **2015/2016** | **2016/2017** | **2017/2018** |
| --- | --- | --- | --- | --- | --- | --- |
| General Universitario de Castellón | X | X | X | X | X | X |
| Universitario de La Plana | X | X | X |  |  |  |
| Arnau de Vilanova |  |  | X |  |  |  |
| Universitario y Politécnico La Fe |  | X | X | X | X | X |
| Universitario Doctor Peset | X | X | X | X | X | X |
| Universitario de La Ribera |  |  | X |  |  |  |
| Universitario San Juan de Alicante | X | X | X |  |  |  |
| General Universitario de Elda | X | X | X |  |  |  |
| General Universitario de Alicante |  |  | X | X | X | X |
| Universitario del Vinalopó |  |  | X |  |  |  |

**Supplementary Table S2.** ICD9 and ICD10 codes according to the International Statistical Classification of Diseases and Related Health Problems 10^th^ Revision of the World Health Organization considered for reporting main and secondary discharge diagnoses.

| **DISEASES** | **ICD9 codes** | **ICD10 codes** |
| --- | --- | --- |
| **CIRCULATORY SYSTEM-RELATED DISEASE** |  |  |
| Hypertensive diseases | 402 | I11, I16 |
| Ischemic heart diseases | 410-414 | I20-I25 |
| Pulmonary heart disease | 415-417 | I26-I27 |
| Other forms of heart disease | 420-425, 427-428 | I30, I31, I33, I38-I42, I46-I50 |
| Cerebrovascular diseases | 430-438 | I63-I67 |
| Diseases of arteries | 444-445, 449 | I74-I76 |
| **RESPIRATORY SYSTEM-RELATED DISEASE** |  |  |
| Acute upper respiratory infections | 460-465 | J00-J06 |
| Influenza/pneumonia | 480-488 | J09-J18 |
| Other acute lower respiratory infections | 466 | J20-J22 |
| Chronic lower respiratory diseases | 490-496 | J40-J47 |
| Other respiratory diseases (interstitium) | 514, 518.82 | J80, J81 |
| Suppurative and necrotic conditions of the lower respiratory tract | 510, 513 | J85-J86 |
| **SELECTED POTENTIAL INFLUENZA-RELATED COMPLICATIONS** |  |  |
| Pneumonia | 480-486 | J12-J18 |
| Heart failure | 428 | I50 |
| Acute myocardial infarction | 410 | I21 |
| Atrial fibrillation | 427.31, 427.32 | I48 |
| Stroke | 434.01, 434.11, 434.91 | I63 |

ICD, international Classification of Diseases

**Supplementary Table S3.** Included subjects and laboratory-confirmed influenza (LCI) cases by season and overall. Hospitalized adults (≥18 years old) from the Valencia Hospital Surveillance Network (VAHNSI), Valencia Region, Spain (2012/2013 to 2017/2018).

|  | **Season 12/13** | | **Season 13/14** | | **Season 14/15** | | **Season 15/16** | | **Season 16/17** | | **Season 17/18** | | **Overall** | |
| --- | --- | --- | --- | --- | --- | --- | --- | --- | --- | --- | --- | --- | --- | --- |
|  | **N** | **%** | **N** | **%** | **N** | **%** | **N** | **%** | **N** | **%** | **N** | **%** | **N** | **%** |
| **Included and tested** | **1632** | **100** | **1817** | **100** | **3456** | **100** | **2108** | **100** | **1645** | **100** | **2887** | **100** | **13545** | **100** |
| **LCI** | 187 | **11.46** | 310 | **17.06** | 673 | **19.47** | 262 | **12.43** | 232 | **14.10** | 593 | **20.54** | 2257 | **16.66** |
| **Influenza** |  |  |  |  |  |  |  |  |  |  |  |  |  |  |
| A(H1N1) pdm09 | 61 | 32.62 | 242 | 78.06 | 9 | 1.34 | 142 | 54.20 | 0 | 0.00 | 112 | 18.89 | 566 | 25.08 |
| A(H3N2) | 5 | 2.67 | 58 | 18.71 | 561 | 83.36 | 2 | 0.76 | 222 | 95.69 | 269 | 45.36 | 1117 | 49.49 |
| A not subtyped | 2 | 1.07 | 10 | 3.23 | 39 | 5.79 | 40 | 15.27 | 10 | 4.31 | 22 | 3.71 | 123 | 5.45 |
| B/Yamagata | 117 | 62.57 | 0 | 0.00 | 60 | 8.92 | 0 | 0.00 | 0 | 0.00 | 182 | 30.69 | 359 | 15.91 |
| B/Victoria | 1 | 0.53 | 0 | 0.00 | 0 | 0.00 | 64 | 24.43 | 0 | 0.00 | 3 | 0.51 | 68 | 3.01 |
| B not subtyped | 1 | 0.53 | 0 | 0.00 | 4 | 0.59 | 14 | 5.34 | 0 | 0.00 | 5 | 0.84 | 24 | 1.06 |

LCI, Laboratory-confirmed influenza

**Supplementary Table S4.** Included subjects (18+) and laboratory-confirmed influenza (LCI) cases by age and overall. Hospitalized adults (≥18 years old) from the Valencia Hospital Surveillance Network (VAHNSI), Valencia Region, Spain (2012/2013 to 2017/2018).

|  | **18-49** | | **50-64** | | **65-74** | | **75+** | | **Overall** | |
| --- | --- | --- | --- | --- | --- | --- | --- | --- | --- | --- |
|  | **N** | **%** | **N** | **%** | **N** | **%** | **N** | **%** | **N** | **%** |
| **Included and tested** | **1380** | **100** | **2084** | **100** | **2803** | **100** | **7278** | **100** | **13545** | **100** |
| **LCI** | 226 | **16.38** | 341 | **16.36** | 511 | **18.23** | 1179 | **16.20** | 2257 | **16.66** |
| **Influenza** |  |  |  |  |  |  |  |  |  |  |
| A(H1N1) pdm09 | 97 | 42.92 | 158 | 46.33 | 126 | 24.66 | 185 | 15.69 | 566 | 25.08 |
| A(H3N2) | 67 | 29.65 | 115 | 33.72 | 255 | 49.90 | 680 | 57.68 | 1117 | 49.49 |
| A not subtyped | 17 | 7.52 | 19 | 5.57 | 22 | 4.31 | 65 | 5.51 | 123 | 5.45 |
| B/Yamagata | 36 | 15.93 | 35 | 10.26 | 75 | 14.68 | 213 | 18.07 | 359 | 15.91 |
| B/Victoria | 5 | 2.21 | 10 | 2.93 | 26 | 5.09 | 27 | 2.29 | 68 | 3.01 |
| B not subtyped | 4 | 1.77 | 4 | 1.17 | 7 | 1.37 | 9 | 0.76 | 24 | 1.06 |

LCI, Laboratory-confirmed influenza
